# Obesity, diet and nutraceuticals

**DOI:** 10.1186/1129-2377-16-S1-A28

**Published:** 2015-09-28

**Authors:** Cherubino Di Lorenzo, Gianluca Coppola

**Affiliations:** Don Carlo Gnocchi Onlus Foundation, Milan, Italy; Department of Neurophysiology of Vision and Neurophthalmology, G.B. Bietti Foundation-IRCCS, Rome, Italy

Migraine is the most prevalent neurological disorder worldwide, affecting on average 12% of the general population. Recent data support migraine as part of the metabolic syndrome (MetSyn) spectrum. The evidence of an association between obesity, insulin resistance, and migraine, all having in common the ability to favor a general pro-inflammatory state, reinforces this view. Under a neuronal point of view, another possible link between migraine and MetSyn is, other than the inflammatory state, the cellular energetic deficit. Indeed, it is well known that a mitochondrial energetic dysfunction may play a role in migraine pathogenesis, that MetSyn could further impair mitochondrial metabolism increasing oxidative stress, and that nutraceuticals involved in mitochondrial oxidative phosphorylation (OXPHOS) metabolism (Riboflavin, CoQ10, and magnesium) can be used as migraine prophylactic treatments. Here we review the evidence of MetSyn and migraine association and possible therapeutic implications.

There is a close relationship between migraine and MetSyn, especially insulin resistance and obesity. More in detail, obesity is regarded as an important factor of chronification for migraineur patients, while weight-loss seems to have a protective effect. The main player of this association seems to be the obesity-related inflammation and an adequate slimming diet can act to reduce this inflammatory mechanism. In fact, inflammatory cytokines are higher in obese subjects and normalized by weight reduction. Moreover, several foods could act as triggers for some patients by an inflammatory antibody mediated pathway, by a direct histamine or other vasoactive peptides effect, or by modifying the quality and quantity of fat intake. Also, altered insulin metabolism could be related to leptins and nitric oxide (NO) stress, both inducing inflammatory effects involved in migraine pathogenesis. Some prophylactic treatments induce increase of weight, insulin and leptins that can counteract the therapeutic effect; it is the “prophylactic paradox”: a long lasting migraine could be worsened by the drug-induced obesity.

Therefore, there is the rationale for the adoption of specific dietetic patterns as complementary treatment in migraine management. In particular, the ketogenic diet is worthy of mention because of its multiple effects on the above-mentioned mechanisms, since it induces a significant weight-loss (when prescribed as very-low calorie diet), normalizes leptins and hyperinsulinemia, improves mitochondrial OXPHOS metabolism, and has a ketone bodies induced anti-inflammatory effect.

In summary, more attention should be paid to metabolic implications of migraine and its treatment to further improve, or at least not worsen, patients’ conditions and reduce migraine-related comorbidities and complications.

